# The Accessory Genome as a Cradle for Adaptive Evolution in Pathogens

**DOI:** 10.1371/journal.ppat.1002608

**Published:** 2012-04-26

**Authors:** Daniel Croll, Bruce A. McDonald

**Affiliations:** Plant Pathology, Institute of Integrative Biology, ETH Zurich, Zurich, Switzerland; Duke University Medical Center, United States of America

Pathogens offer many of the most fascinating and well-studied examples of evolution because their speed of adaptation allows observation of evolutionary change within human lifetimes. The rapid evolution observed in pathogenic viruses, bacteria, and fungi affect human, animal, and plant health globally. For example, influenza viruses regularly recombine genes affecting host range, infection pathways, and virulence prior to the emergence of deadly outbreaks [1]. The emergence of antibiotic resistance in bacteria, including highly resistant *Staphylococcus aureus* strains and the re-emergence of resistant tuberculosis, is well documented [2], [3]. The recent outbreak in Africa of wheat stem rust caused by the Ug99 pathotype of the fungus *Puccinia graminis tritici* surprised many plant pathologists, who thought this ancient pathogen was defeated during the green revolution [4]. It is clear that pathogens engage host species in a constant race to evolve new defense mechanisms. By extension, we are constantly challenged to find new treatments and better containment measures to protect human health and essential crops.

## Is There a Genetic Mechanism Enabling More Rapid Evolution in Pathogens?

Extensive work on the molecular basis of pathogen adaptation reveals several common themes. Pathogens usually have higher rates of reproduction and shorter generation times than their hosts. Large effective population sizes maintain genetic diversity while recombination or reassortment puts together favorable combinations of mutations. Switches in reproductive modes are frequent, as epidemics are often caused by an emerging clone that has fixed a favorable combination of alleles and gained the potential for rapid dissemination. However, the astonishing capacity of pathogens to evolve rapidly is not based solely on these life-history traits.

It appears that rapid adaptation to hosts and new environments is favored when pathogens carry a highly variable, rapidly mutating genomic compartment. For example, viruses can carry satellite RNAs [5] while toxins and antibiotic resistance factors are often encoded on plasmids in bacteria [6]. Pathogenic fungi often possess highly variable regions that may be “lineage-specific” (i.e., restricted to a set of isolates) and cover entire chromosomes. Such chromosomes have been called accessory, supernumerary, B, or dispensable chromosomes. Their discovery dates back to 1907, with the very first observation of unusual chromosomes (in hemipteran insects) [7]. We think that the term “accessory” provides the most neutral description of these chromosomes and the term is well aligned with the literature on bacterial pathogens. The rapidly evolving compartments of pathogen genomes have been associated with high mutation rates, the acquisition of foreign genes, copy number polymorphisms, and frequent ectopic recombination [8]–[11]. By comparison, the core genome carries essential genes that encode basal functions such as cellular metabolism and reproduction. Because of its essential functions, the core genome is thought to be shielded from high recombination and mutation rates and insertion of novel genes. Recent genomic comparisons have revealed that many pathogens share a strikingly compartmentalized genome, which we will refer to as the “2-speed genome” following Raffaele et al. [10].

## Fungal Pathogens Carry an Extensive Set of Accessory Genomic Elements

Fungi possess sophisticated accessory genomic elements linked to pathogenicity that can span entire chromosomes. We will focus this review on these accessory chromosomes.

*Nectria haematococca* was the first discovered and probably best-studied fungus carrying accessory chromosomes. Accessory chromosome 14 carries a cluster of genes encoding detoxification of a phytoalexin produced by pea plants, enabling isolates carrying this chromosome to be virulent on peas [11], [12]. Chromosome 14, as well as two other accessory chromosomes (15 and 17), show striking differences to the core chromosomes. A substantial fraction of the genes located on these chromosomes are more similar to the distantly related *Aspergillus* clade than to the more closely related *Fusarium* species [11]. These accessory chromosomes are also enriched in transposable elements and gene duplications. The origin of *N. haematococca* accessory chromosomes remains unresolved, as it is unknown whether the chromosomes originated from ancient core chromosomes or whether interspecific transfers might have been involved [11].

A similar picture has emerged of the accessory chromosomes of the tomato pathogen *Fusarium oxysporum* (*f. sp. lycopersici*). Nearly 40% of the *F. oxysporum* genome was specific to the tomato-infecting lineage and lacked synteny with closely related species [13]. In this study, accessory chromosomes contained the majority of transposable elements and genes were enriched in secreted proteins and virulence factors. Similar to what was found for *N. haematococca*, phylogenetic analyses of the genes on *F. oxysporum* accessory chromosomes point to horizontal transfer as the most likely origin. The consensus for a broad set of genes located on the accessory chromosomes points to an emergence coinciding with the root of the *F. oxysporum*, *F. verticillioides*, and *F. graminearum* clade but prior to the split with *N. haematococca*. Interestingly, *F. oxysporum* chromosome 14 can be exchanged among different strains despite the lack of sexual recombination [13].

**Figure 1:**
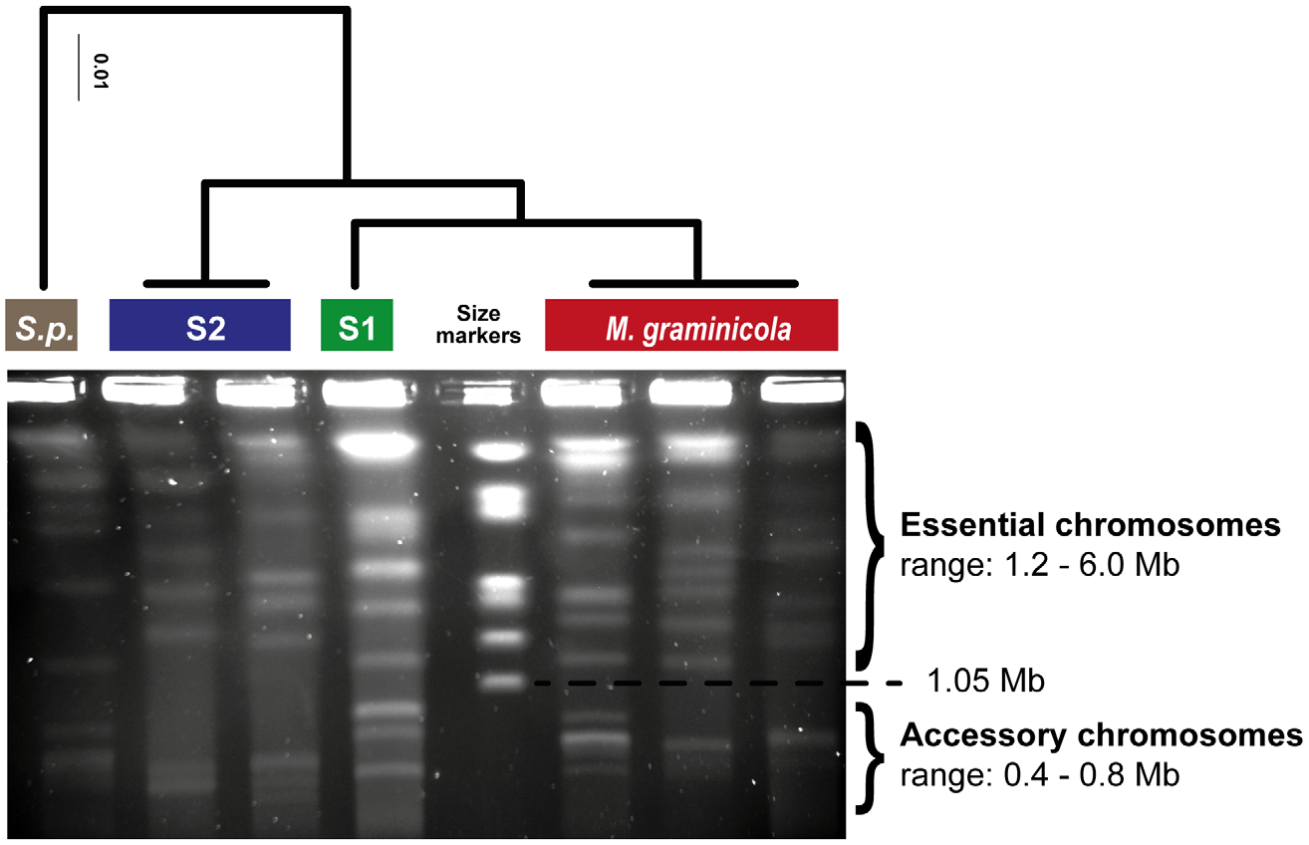
Diversity in essential and accessory chromosomes in the *Mycosphaerella* clade. Chromosomes of the outgroup species *Septoria passerinii* (S.p.), two isolates of *Mycosphaerella* S2, one isolate of *Mycosphaerella* S1, and three isolates of *M. graminicola* were visualized by pulsed-field gel electrophoresis (PFGE). *Mycosphaerella* S2 and S1 are found on wild grasses in Iran, the center of origin of *M. graminicola*, a major leaf pathogen of wheat [14]. The size markers are *Hansenula wingei* chromosomes. The phylogenetic tree is based on a six-gene alignment.

*Mycosphaerella graminicola*, a major wheat pathogen that originated in the Fertile Crescent during the domestication of wheat, carries an especially rich set of accessory chromosomes [14], [15] (Figure 1). *M. graminicola* maintains extensive chromosomal length polymorphism within populations, likely through the high degree of chromosomal rearrangements occurring during meiosis [8], [16]. Similar to other fungi, the genes located on accessory chromosomes show only weak homology to genes found on core chromosomes or closely related species. The *Mycosphaerella* clade may provide an interesting model for the evolution of accessory chromosomes as closely related sister species carry sets of chromosomes of similar length. However, it is currently unknown whether these chromosomes are accessory or part of the core chromosome set. Interestingly, genes on accessory chromosomes with homologs in sister species showed an accelerated rate of evolution compared to the core genome [17].

## The Two-Speed Genome as a Fundamental Characteristic of Rapidly Evolving Pathogens

This brief survey of fungal pathogens suggests that at least some portions of the accessory chromosomes constitute the rapidly evolving segment of a genome that evolves at different speeds. Compartmentalization of the genome may provide several evolutionary advantages to the pathogen. Among the benefits of an accessory genome are the potential to acquire novel virulence factors that can expand host range. A novel gene function may evolve through gene or segmental duplication within the genome or through horizontal transfer. Depending on the mechanism of gene insertion or duplication, the novel gene may disrupt an existing, functional gene, influence gene transcription, and/or favor non-homologous recombination. The higher rate of substitutions and non-homologous recombination may be permissive only for chromosomes largely devoid of essential genes. Accessory chromosomes, therefore, provide an ideal “testing ground” for the evolution of new genes. A pathogen faced by a high heterogeneity among potential hosts may benefit from having virulence genes located on accessory chromosomes. A gene that is beneficial to the pathogen only on a particular host genotype but detrimental on other hosts may more rapidly increase or decrease in frequency in a population if it is located on an accessory chromosome.

A high mutation rate coupled with the expected lack of homologous pairings during meiosis for an accessory chromosome may lead to gradual decay of its chromosomal structure. Similar to the processes observed for the mating type loci in *Cryptococcus neoformans* and *Neurospora tetrasperma* [18], [19], as well as heteromorphic animal sex chromosomes [20], an accessory chromosome is expected to gradually decay in a population unless homologous pairings help purge deleterious mutations. This process, termed Muller's ratchet, was proposed to operate on animal accessory chromosomes [21]. In fungal pathogens, large population sizes are expected to slow a ratchet process of degeneration. However, as many genes on accessory chromosomes are not likely to be under strong selection pressure, segmental deletions could be fixed either through founder events or selective sweeps affecting the pathogen populations. Hints of the decay of accessory chromosomes in fungi are found in *M. graminicola*, where several accessory chromosomes show large length variation segregating within populations [16]. A largely unexplored aspect of accessory chromosomes is their capacity for meiotic drive (i.e., a transmission rate higher than expected under Mendelian segregation). The extensive literature on animal accessory chromosomes shows that meiotic drive is ubiquitous [22]. The propensity of accessory chromosomes to be horizontally transferred under lab conditions in *F. oxysporum* and *Alternaria alternata* [13], [23] may provide an analogous scenario to “selfish” drive transmission. Understanding the evolutionary dynamics of accessory chromosomes in fungi will require consideration of both the beneficial effects of virulence genes located on these chromosomes and their transmission characteristics through both meiosis and horizontal chromosome transfer.

## Toward a More Comprehensive Understanding of Accessory Genomes in Pathogens

Recognizing common themes in the vast diversity of organisms carrying accessory genome elements enables us to refine our understanding of the genetic basis of rapid evolution in pathogens. But a central question remains largely unanswered: How are accessory genomic elements generated and how are they maintained through evolution?

In bacteria, important virulence traits and antibiotic resistance are often encoded on accessory genome elements such as plasmids. The potential for the evolution of antibiotic resistance and increased virulence is often directly related to the mobility of the genes encoding these traits among strains. Will similar trends emerge for the fungal accessory genomes? Do fungal clades with a propensity to carry accessory chromosomes (such as *Fusarium* or *Mycosphaerella*) pose a more significant threat for rapid evolution of virulence or drug resistance?
